# Operating Characteristics of High-Order Distributed Feedback Polymer Lasers

**DOI:** 10.3390/polym11020258

**Published:** 2019-02-03

**Authors:** Puxi Zhou, Lianze Niu, Anwer Hayat, Fengzhao Cao, Tianrui Zhai, Xinping Zhang

**Affiliations:** 1 Institute of Information Photonics Technology and College of Applied Sciences, Beijing University of Technology, Beijing 100124, China; niulianze@126.com (L.N.); anwerhayatnoor@gmail.com (A.H.); wincfz@163.com (F.C.); zhangxinping@bjut.edu.cn (X.Z.); 2 Department of Electrical Engineering and Computer Science, Samueli School of Engineering, University of California Irvine, Irvine, CA 92697, USA; puxiz@uci.edu

**Keywords:** polymer lasers, high-order, laser threshold, slope efficiency

## Abstract

In this study, high-order distributed-feedback (DFB) polymer lasers were comparatively investigated. Their performance relies on multiple lasing directions and their advantages include their high manufacturing tolerances due to the large grating periods. Nine laser cavities were fabricated by spin-coating the gain polymer films onto a grating structure, which was manufactured via interference lithography that operated at the 2^nd^, 3^rd^, and 4^th^ DFB orders. Low threshold lasing and high slope efficiency were achieved in high-order DFB polymer lasers due to the large grating groove depth and the large gain layer thickness. A high-order DFB configuration shows possible advantages, including the ability to control the lasing direction and to achieve multiple-wavelength lasers. Furthermore, our investigation demonstrates that the increase in threshold and decrease in slope efficiency with an increase in the feedback order can be limited by controlling the structural parameters.

## 1. Introduction

Fluorene-based polymers, which have large stimulated emission cross-sections and wide gain spectra ranges, are ideal active materials for creating distributed feedback (DFB) lasers [1,2,3]. DFB lasers have attracted extensive attention due to their promising performance regarding mode selection and operational stability. Hence, DFB polymer lasers are a good platform for investigating how structural parameters impact laser operational properties [4,5,6,7,8]. In the realm of DFB configurations, the 1^st^ and 2^nd^ order laser cavities have already been comprehensively studied [9,10,11,12], especially 2^nd^ order DFB lasers, which has a feedback mechanism that was found to be provided by 2^nd^ order diffraction. In contrast, 1^st^ order diffraction facilitates the mode selection and the output coupling [9,11]. Conversely, higher-order DFB lasers have only been the subject of some basic investigations because of their relatively low coupling efficiency [13,14,15], which indicates a weak feedback mechanism and thus, a higher lasing threshold compared to lower-order DFB lasers. Recently, some interesting results have been reported in the high-order DFB polymer lasers. High-order DFB configurations were adopted for designing low-cost laser cavities [16], controlling laser output directions [17], and achieving multiple-wavelength or broad-range-wavelength lasers [18,19,20,21,22,23]. Obviously, higher-order DFB cavities have advantages during the fabrication process, which are mainly due to their requirements of larger grating periods. Indeed, for 1^st^ and 2^nd^ order DFB lasers, optical gratings that are strictly sub-wavelength are needed. As the Bragg order increases, based on the Bragg condition 2$n_{eff}$ *Λ* = mλ (in which $n_{eff}$ is the modal effective refractive index, *Λ* is the grating period, m is the Bragg order, and λ is the free-space lasing wavelength), the optical grating period will increase correspondingly. Hence, the requirements for high resolution fabrication are reduced [18,24]. The above Bragg condition states that one may continue operating at a given wavelength by increasing the period *Λ* and by resorting to selecting a feedback mechanism associated with a higher order m. However, working at a higher order also results in some coupling (i.e., feedback intensity) disadvantages, resulting in a higher lasing threshold. On the other hand, as shown in this paper, the gain thresholds and slope efficiencies of DFB polymer lasers can also be refined via adjusting structural parameters, which are mainly the grating groove depth of the grating structure and the gain layer thickness. For a 2^nd^ order cavity, these effects have been well-discussed [4,5]. However, comparative investigations are required for higher-order DFB polymer lasers. In this present study, we focus on improving the performance of high-order DFB polymer lasers, especially aiming to improve the lasing threshold and the slope efficiency.

In this article, we experimentally and theoretically investigate the performance of high-order DFB structures. By spin-coating the gain polymer onto the grating structures, the total cavity heights are in the order of hundreds of nanometers and the guiding structures only support the fundamental propagating mode (TE_0_). One-dimensional DFB polymer lasers of the 2^nd^ and 4^th^ order have been achieved under optical pumping. The laser properties related to surface emission, such as thresholds and slope efficiencies, have been extracted. A comparison of the experimental performances has been made based on different Bragg orders and various structural parameter combinations in the DFB polymer lasers. In general, a laser’s performance deteriorates when the Bragg order is increased, while a larger grating groove depth and larger gain layer thickness will decrease the lasing threshold and improve the slope efficiency.

## 2. Principle, Design, and Fabrication

### 2.1. Output Direction Analysis

**Figure 1:**
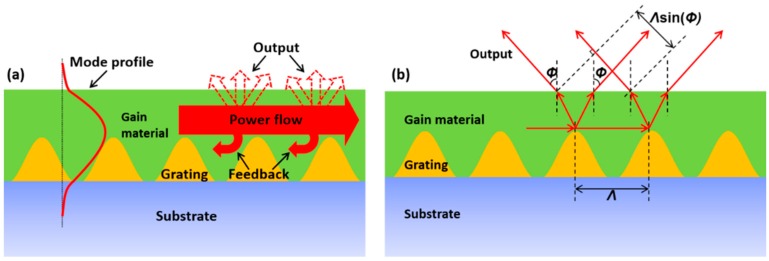
(**a**) Illustration of the propagating mode profile as well as the feedback and output mechanism. (**b**) Optical path analysis for interference output rays of a high-order distributed-feedback (DFB) laser.

A DFB cavity provides a distributed feedback mechanism and output coupling through a selected diffraction order of an optical grating, which depends on the Bragg order of the DFB lasers. For a 1^st^ order DFB laser, both feedback and output mechanisms are supported by 1^st^ order diffraction. As stated before, for a 2^nd^ order DFB laser, the feedback mechanism and output coupling are provided by 2^nd^ and 1^st^ order diffraction, respectively. In general, for a $m^{th}$ order DFB laser (*m* > 2), the feedback mechanism is always established via $m^{th}$ order diffraction, whereas various output coupling phenomena can occur via different diffraction orders with order numbers below or equal to |*m*|. In turn, this may also lead to the multiple output directions observed during laser operation. The laser output that resulted from $m^{th}$ order diffraction is known as the edge emission, with the output beam along the direction ϕ=π/2 (see Figure 1b). The laser output obtained from other diffraction orders is known as the surface emission (ϕ∈[0,π/2)). In the following section, we will develop an equation that directly relates the surface emission directions with the Bragg order of the DFB lasers.

To achieve this, we conducted an analysis (as illustrated in Figure 1) of high-order DFB lasers with the cavity configuration adopted in this paper. The Bragg condition is $2n_{eff}\Lambda =m\lambda$, where the modal effective refractive index $n_{eff}$ is dependent on the mode profile of the propagating mode, which is schematically drawn on the left side of Figure 1a. On the right side of Figure 1a, the output coupling is illustrated, where dashed arrows indicate the lasing output directions that may occur. In Figure 1b, red arrows represent the propagating and emitted light rays.

To form an emission beam along a direction ϕ, the emitted light rays should satisfy the condition of constructive interference, which is described as follows:

(1) $$\frac{2\pi n_{eff}}{\lambda }\cdot \Lambda +\frac{2\pi }{\lambda }\cdot \Lambda sin\phi =l\cdot 2\pi$$

where $n_{eff}$ is the modal effective refractive index, *Λ* is the grating period, and λ is the free-space lasing wavelength, ϕ is the angle of the laser emission, l is a positive integer. After this, if we implement the transform $\Lambda =\left(m\lambda \right)/\left(2n_{eff}\right)$, the equation that directly relates the Bragg order number with the emission angle ϕ is obtained as:

(2) $$m\cdot \left(1+\frac{sin\phi }{n_{eff}}\right)=2l,\;\;\phi \in \left[0,\frac{\pi }{2}\right),$$

where *m* is the Bragg order, ϕ is the angle of the laser emission, $n_{eff}$ is the modal effective refractive index, l is a positive integer. The emission angle defines the output direction as illustrated in Figure 1b.

### 2.2. Cavity Design and Sample Fabrication

Equation (2) states that for even-order DFB lasers (i.e., with *m* = 2, 4, 6, etc.), the vertical-surface emission is always supported because there always exists an m=2l (where m is the Bragg order, l is a positive integer) so that ϕ= 0° is a solution of Equation (2). At the same time, for DFB lasers with a Bragg order number that is larger than 2 (i.e., with m = 3, 4, 5, etc.), at least one pair of symmetric slanted emissions can be generated. In the following section, 3^rd^ and 4^th^ order DFB polymer lasers are selected to be representative of odd- and even-order DFB lasers and we explore the far-field pattern and the impact of the structural parameters on their operational properties.

**Figure 2:**
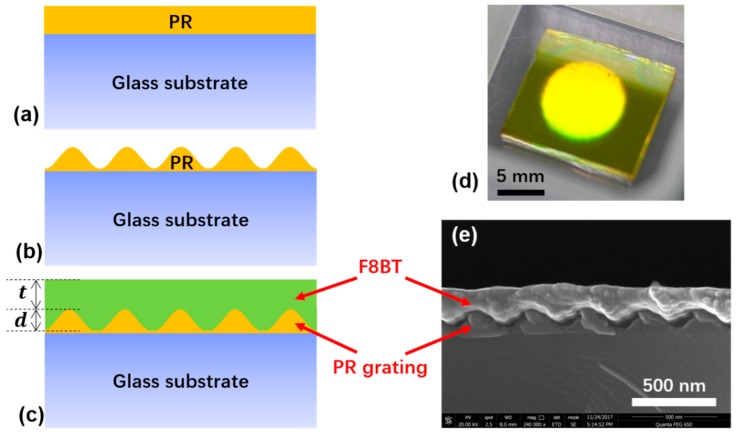
Panels (**a**)–(**c**) detail the fabrication process of the DFB polymer laser. (**a**) Spin-coat photoresist onto glass substrate. (**b**) Fabricate the grating structure by interference lithography. (**c**) Spin-coat polymer on top of the grating, where *t* is the gain layer thickness and *d* is the grating groove depth. (**d**) Photograph of fabricated sample. (**e**) SEM image of the cross-section of the sample showing the photoresist (PR) grating profile and the gain polymer.

As shown in Figure 2, a classical configuration of a DFB resonator was implemented that provides a strong modulation on the gain material thickness, effective refractive index and optical gain. This may help to compensate for the weakness of the coupling efficiency in high-order DFB polymer lasers. To fabricate the grating structures, as shown in Figure 2a,b, a photoresist (PR, AR-P3170, Strausberg, Germany) was first spin-coated onto a glass substrate to form a thin film. The thickness of the thin film was controlled by changing the spin-coating speed. The film thickness decreases with increasing the spin-coating speed, and vice versa. After this, the prepared sample was exposed for interference lithography using a 343-nm laser beam (Flare NX Laser, coherent, Santa Clara, CA, USA). After exposure, the grating structures were fabricated by using a developer (AR-300-47, Allresist, Strausberg, Germany) for a few seconds to dissolve the imprinted parts. The exposure and dissolving time were adjusted along with the various structural parameters so that the grating groove depth *d* was almost equal to the PR film thickness while this also ensured a well-shaped sinusoidal grating. The grating period Λ was determined from Λ=λ/(2sinθ), where λ= 343 nm is the wavelength used in the lithographic process and θ is the angle between the lithography interference beams [25,26]. By tuning the angle θ, a wide range of grating periods could be obtained. Finally, the fluorene-based polymer, which was namely poly[(9,9-dioctylfluorenyl-2,7-diyl)-altco-(1,4-benzo(2,1′,3)-thiadiazole)] (F8BT, American Dye Source, Monteral, QC, Canada), was dissolved in xylene at a concentration of 23 mg/ml and spin-coated onto the grating structure. The gain layer thickness *t* was also controlled by adopting different spin-coating speeds [26]. Figure 2c depicts a schematic diagram of a high-order DFB structure with separately controlled grating groove depth *d* and gain layer thickness *t*. Table 1 summarizes the spin-coating speeds needed to achieve specific structural parameters. In Figure 2, the panels (d) and (e) display the fabricated laser sample and scanning electron microscopy (SEM, Hitachi S-4800, Tokyo, Japan) images of the laser’s cross-section, respectively.

To enhance the coupling efficiency of high-order Bragg resonators and achieve a DFB polymer laser with a low lasing threshold, we first optimized the grating groove depth *d* [13]. Larger grating groove depths strengthen the index and gain modulation, establish a stronger feedback mechanism and lower the lasing threshold. Additionally, it is worth noting that larger grating groove depths also enhance the diffraction effect, through which the output coupling is strengthened. As a result, the laser system would suffer an increment of cavity loss [4]. This effect may cancel out the performance improvement that arises from the feedback enhancement. To compensate for this extra “loss”, we can increase the gain layer thickness *t* along with *d*, which will improve the average gain. In this regime, the average gain is defined as the average amount of gain that is experienced by the propagating modes during one trip along the cavity. Here, we reiterate our goal that high-order DFB polymer lasers with optimized operational properties can be achieved by adjusting the structural parameters. Since 2^nd^ order (*m* = 2) DFB polymer lasers have been widely adopted due to their promising performance, we used 2^nd^ order lasers as a benchmark to assess the performance of the high-order (3^rd^ and 4^th^) DFB polymer lasers that were investigated in this present study. Specifically, based on our analysis, we set the structural parameter combinations (*d* [nm], *t* [nm]) for three 2^nd^ order DFB polymer lasers to be: (120, 150), (150, 170), and (170, 190). The three 3^rd^ order DFB polymer lasers were fabricated with the same *d* and *t* parameters as used for the 2^nd^ order lasers. In the three fabricated 4^th^ order DFB polymer lasers, larger grating groove depths and larger gain layer thickness were adopted compared with to the 2^nd^ and 3^rd^ order lasers, with the impact of each parameter inspected separately. The parameter combinations for the three 4^th^ order DFB polymer lasers were (190, 220), (190, 260), and (220, 260).

It is important to note that the optical gain is a function of the wavelength and that the amount of gain influences operational properties [2]. Hence, the grating periods of the DFB cavities investigated here were carefully adjusted for each laser sample to ensure that they operated in a narrow wavelength range where the optical gain was approximately constant. Furthermore, the effective refractive index of the resonating mode in the cavity increases with an increase in the grating groove depth and gain layer thickness [27]. Thus, by decreasing the grating period, one can keep the lasing wavelength relatively constant according to the Bragg condition. As a result, a more valid comparison between the various laser samples is ensured. The dimensions for all nine fabricated cavities are summarized in Table 2.

## 3. Experiment and Discussion

**Figure 3:**
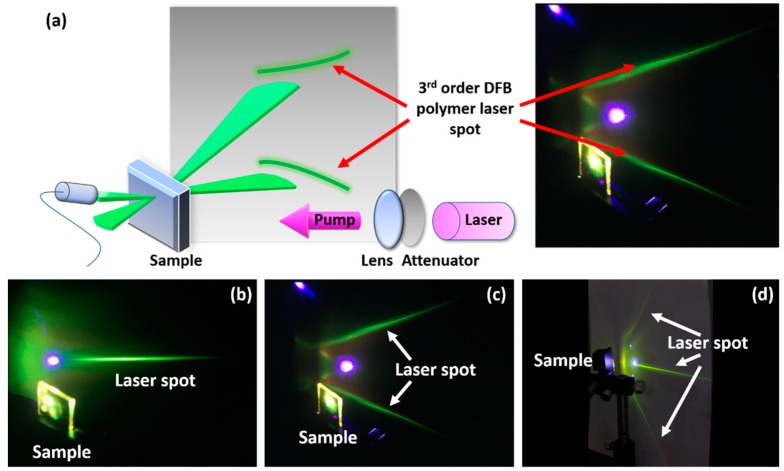
(**a**) Illustration of the experimental setup and formation mechanism of the pattern of a 3^rd^ order DFB polymer laser. The purple spots shown in the right photograph are the reflection and diffraction of the pumping laser. (**b**) 2^nd^ order laser pattern. (**c**) 3^rd^ order laser pattern. (**d**) 4^th^ order laser pattern.

The prepared laser samples are pumped by a frequency-doubled Ti:sapphire laser operated at a wavelength of 400 nm with pulse widths of 200 fs and repetition frequency of 1 kHz. As shown in Figure 3a, the pumping power is tuned via a neutral density filter and focused on the laser sample after being converged by a convex lens (purple beam). The 3^rd^ order DFB polymer laser was used here as an example. The gray background indicates the screen on which the two green symmetric line-shape patterns were formed from the 3^rd^ order laser emissions, which was illustrated here by the two green fan-shaped beams in front of the laser sample. We recalled that third-order lasers generate a pair of radiated beam directions. Fan-shaped beams are formed because the light confinement happens only along the direction that is orthogonal to grating lines [17].

Since there is no confinement that is parallel to the grating lines, multiple modes with various wavevector components along the grating-line direction are allowed inside the cavity, which results in the fan-shaped emission beam that is always observed for one-dimensional DFB lasers. The two purple spots (right panel) originate from the reflection and grating diffraction of the pumping beam, as denoted by the purple arrows. On the backside of the laser sample, the spectrometer was placed in the normal plane of the laser samples, directly facing the laser emission to record the output spectra. Additionally, the emission angle can be determined by measuring the angle between the spectrometer and the normal of the sample.

The 2^nd^, 3^rd^, and 4^th^ order DFB polymer laser patterns are shown in Figure 3b–d. The 2^nd^ order laser only exhibits vertical surface emission, as explained in Section 2, which results in a single green line-shape pattern on the screen in Figure 3b. The 3^rd^ order laser shows a pair of symmetric patterns while the 4^th^ order laser has not only a vertical emission pattern, but also a pair of slanted emission patterns. The emission angle ϕ with respect to the normal of the 3^rd^ order laser (sample E) was measured to be 33°. The emission angle ϕ with respect to the normal of the 4^th^ order laser (sample I) was measured to be 58°, otherwise it was ϕ = 0°. When these values are inserted into Equation (2), we determined that the modal effective refractive indices were 1.634 and 1.696 for samples E and I, respectively. Additionally, according to the Bragg condition, since both the free space lasing wavelength and grating period are known, the effective refractive indices estimated based on the emission wavelength are 1.65 for sample E and 1.703 for sample I. Thus, the results are in good agreement with each other.

**Figure 4:**
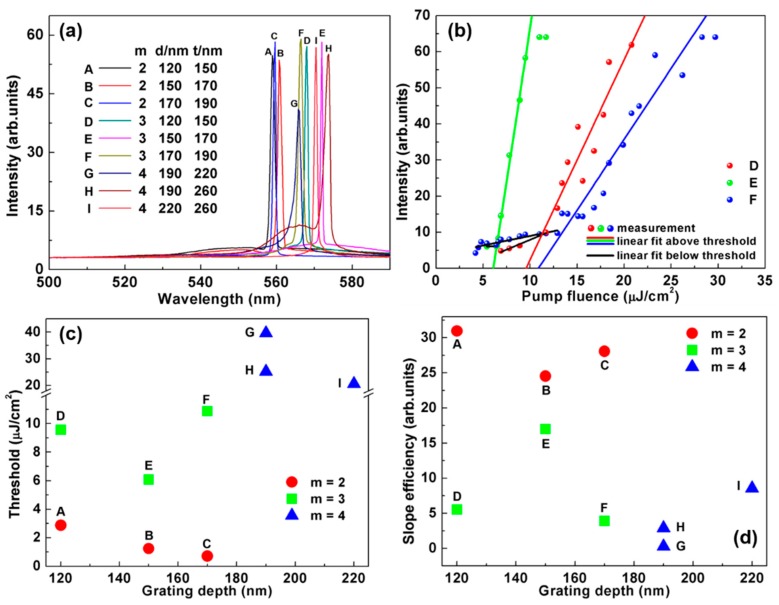
(**a**) Spectra of 9 laser samples; (**b**) measurement points and linear fits for the 3^rd^ order DFB polymer laser; (**c**) threshold plot for 9 laser samples; and (**d**) slope efficiency plot for 9 laser samples.

The emission spectra of the nine investigated laser samples are plotted in Figure 4a. The emission wavelengths were purposely constrained in the range of 559–574 nm by adjusting the grating period as explained earlier with a relatively constant material optical gain. Therefore, when comparing the performance among the laser samples, the influence of the material optical gain on the laser thresholds and the slope efficiencies can be ignored. In Figure 4b, the laser output intensities of the three 3^rd^ order laser samples are plotted as a function of the pumping fluence. The lasing thresholds of sample D, E and F are 9.6, 6.1, and 10.9 $\mu J/cm^{2}$, respectively, which are similar to the the lasing thresholds of the other reported low-threshold polymer lasers [28,29]. Additionally, it can be seen in Figure 4b that after a specific pumping fluence, the output intensity of each laser sample remains constant, which represents that the lasers have reached saturation.

Figure 4c shows the threshold characterizations for the nine laser samples labeled with their grating groove depth *d* and Bragg order *m*. It is clear to see that the laser thresholds increase with the Bragg order number, which is attributed to the decayed coupling efficiencies [13]. We also found that the increment of grating groove depth and gain layer thickness will lead to a decrease in the lasing thresholds, except for sample F. As expected, a larger grating groove depth provides a strengthened feedback mechanism, which results in a reduction in the lasing threshold. A thicker gain material layer improves the average gain, which also remarkably decreases the laser thresholds. As for the sample F, the increment of threshold with respect to sample E may be due to the increased output loss, as explained earlier, or from the undesired mode competing or manufacturing defections [27].

In Figure 4d, the slope efficiencies for the nine laser samples are shown with labeled grating groove depth *d* and Bragg order number *m*. From this figure, a trend can be seen as the slope efficiencies decrease with the Bragg order number and the change of the cavity structure can significantly influence the slope efficiencies. Finally, we focused on the operational performances of the three 4^th^ order DFB polymer lasers that are shown in Figure 4c,d (labeled by the blue triangles). After comparing sample G and H, we found that an increase in the gain layer thickness can both reduce the lasing thresholds and enhance the slope efficiencies due to the increased averaged gain. Meanwhile, when inspecting the sample H and I, it was found that an increase in the grating groove depth remarkably improves the slope efficiency because of an enhanced feedback mechanism although it only results in a small reduction in the threshold due to the co-existence of increased output loss.

## 4. Conclusions

High-order DFB polymer lasers were implemented and their performances were systematically investigated. Increasing the Bragg order associated with the feedback mechanism has the advantage of larger grating periods, but also leads to increased laser thresholds and decreased slope efficiencies due to the decreased coupling efficiency of the DFB structure. However, these disadvantages can be ameliorated by adjusting the grating groove depth and the gain layer thickness in the high-order DFB polymer lasers. A larger grating groove depth can provide a stronger feedback mechanism, which gives rise to a remarkable reduction in the lasing threshold and enhances the slope efficiency as shown in this paper. This forms a feedback mechanism related to Brag orders of *m* = 2,3 and 4. A larger gain layer thickness effectively improves the performance due to an increased average gain.

## Figures and Tables

**Table 1:** Chart for spin-coating speeds and corresponding structural parameters.

Material	Photoresist					F8BT				
***d or t*** [*nm*]	120	150	170	190	220	150	170	190	220	260
***krpm***	2.5	2.2	2	1.8	1.6	1.5	1	0.8	0.6	0.4

**Table 2:** Design parameters for the nine DFB polymer laser cavities.

Sample	A	B	C	D	E	F	G	H	I
***m***	**2**			**3**			**4**		
***d*/*nm***	120	150	170	120	150	170	190	190	220
***t*/*nm***	150	170	190	150	170	190	220	260	260
***Λ*/*nm***	355	350	345	530	520	515	675	685	670
